# Epigenetic Mechanism of Heart Failure with Preserved Ejection Fraction (HFpEF)

**DOI:** 10.3390/jcdd13100489

**Published:** 2026-09-29

**Authors:** Suresh C. Tyagi, Alexander V. Ovechkin

**Affiliations:** 1 Department of Physiology, School of Medicine, University of Louisville, HSC-A-1115, 500 South Preston Street, Louisville, KY 40202, USA; 2 Department of Neurological Surgery, School of Medicine, University of Louisville, 220 Abraham Flexner Way, Suite 1512, Louisville, KY 40202, USA; 3 Kentucky Spinal Cord Injury Research Center, School of Medicine, University of Louisville, Louisville, KY 40202, USA

**Keywords:** DNMT, TET, writer, eraser

## Abstract

Heart failure with preserved ejection fraction (HFpEF) is a primary cause in patients with metabolic dysfunction and hypertension. A chronic high fat diet (HFD) creates metabolic dysfunction and dysregulation of epigenetic folate 1-carbon metabolism (FOCM), elevating the homocysteine levels (Hcy, characteristic of homocystinuria or HHcy). Homocystinuria has been associated with an increase in blood pressure (via abrogation eNOS pathways) and hypertension. It is unclear whether HFD and homocystinuria contribute synergistically to HFpEF. The HFD may increase the method of transportation via the SLC25A transporter and dysregulate FOCM, creating homocystinuria. The inhibition of SLC25A by suramin could attenuate the FOCM, DNMT1, and TET2 levels, and cardiac dysfunction during HFD and homocystinuria. Because HFD increases methionine and decreases homocysteine transsulfuration via the SLC7A transporter, we hypothesize that the induction of SLC7A will increase homocysteine transport into the mitochondrial transsulfuration, and will produce H_2_S during HFD and homocystinuria. The overall hypothesis to be tested is that a HFD increases methionine transport by SLC25A and induces epigenetic FOCM, homocystinuria, and decreases mitochondrial transsulfuration by SLC7A, CBS/CSE/3MST and H_2_S, causing HFpEF. This review introduces a novel framework to understand how diet-induced epigenetic disruptions contribute to HFpEF through altered homocysteine metabolism and hypertension. Additionally, it explores the therapeutic potential of SLC25A inhibition via suramin to counteract these effects. Insights gained from those studies will underscore the critical role of HFD-induced HFpEF and open pathways for new interventions.

## 1. Introduction

Heart failure with preserved ejection fraction (HFpEF) and heart failure with mid-range ejection fraction (HFmrEF) are recognized risk factors for cardiac-related deaths, without any concrete treatment strategy for this heart disease burden.

Aligned with the 2026 ESC Guidelines for the management of heart failure, the HFmrEF category has been removed; the current phenotypes are HFrEF (LVEF < 50%) and HFpEF (LVEF ≥ 50%). The current treatment by steroidal or non-steroidal mineralocorticoid receptor antagonists (MRA) for HFpEF has not produced significant outcomes. The contemporary guidelines and consensus documents now support SGLT2 inhibitors in HFpEF/HFmrEF, with strong evidence for a reduction in heart failure events [1].

Epidemiologically, one of the causes of HFpEF is diet-induced metabolic syndrome. In a high fat diet (HFD), especially with red meat, methionine is the dominant amino acid, and its metabolism via epigenetics instigates cardiac fibrosis, mitochondrial bioenergetics pathways through gut dysbiosis and the methionine and homocysteine SLC25A/SLC7A transporters, respectively. Pharmacologically, suramin inhibits SLC25A. The role of volume retention by the kidney during HFpEF is indexed by the biomarker neutrophil gelatinase associated lipocalin (NGAL). Therefore, it is important to discuss the clinical background of HFpEF in relation to diet, methionine, and one-carbon metabolism as well as homocysteine and vascular/cardiac remodeling in conjunction with epigenetic mechanisms in HFpEF, mitochondrial sulfur metabolism via transsulfuration, and H_2_S generation. The SLC25A/SLC7A transporters are the experimental targets, with therapeutic implications and limitations.

Because HFpEF is associated with double or triple hits, such as obesity, hypertension, diabetes, aging, and others, it is obvious that dietary components, along with the above hits, may contribute to this devastating condition. We focus on metabolic dysfunction and hypertension contribution to HFpEF because a diet rich in methionine can cause disruptions in rhythmic methylation/de-methylation and epigenetic control of gene regulation. A typical diet containing 100 g of red meat contains approximately 1 g of methionine. For a single amino acid, this amount is very high. Methionine is the central part of the epigenetic regulation of DNA, RNA, and protein methylation. Rhythmic methylation/demethylation is a constitutive footprint of gene imprinting during development or diseases. The transfer of a methyl group from methionine to DNA/RNA/histone unequivocally creates homocysteine. This homocysteine needs to recycle back to methionine, otherwise it will create neuronal defects, including neural tube defects, which is why folic acid is prescribed during pregnancy.

**Figure 1:**
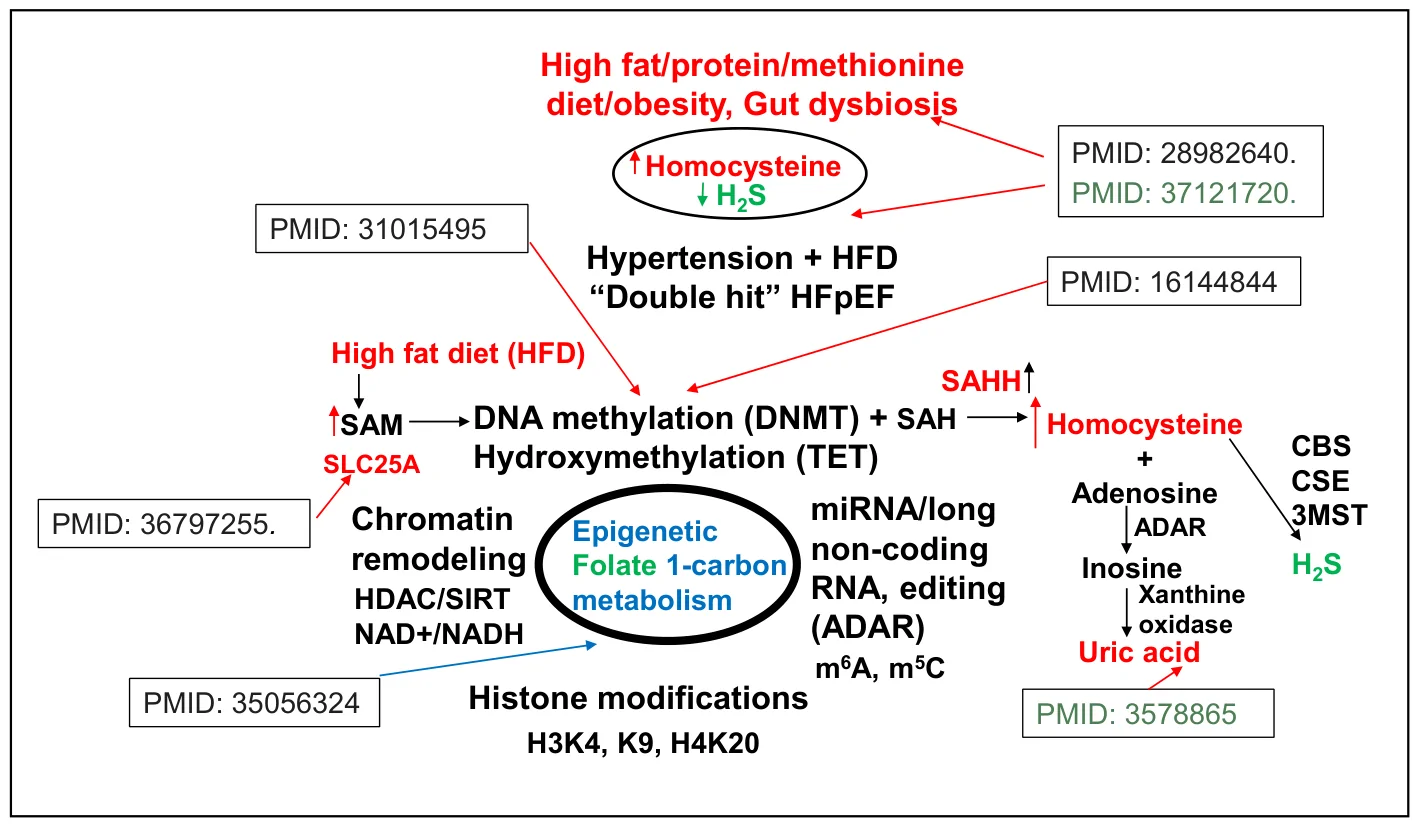
The overall schematic of epigenetics and diet-induced obesity, gut dysbiosis, hypertension and HFpEF: (1) chromatin acetylation/deacetylation, (2) folate 1-carbon metabolism (FOCM), and (3) miRNA, generating uric acid. The sulfur transsulfuation pathways producing H_2_S from homocysteine. To clarify the concept and pathways of each step, the supporting references are reported. The relationship between miRNA/ADAR and uric acid is particularly interesting because SAH is hydrolyzed by SAHH to homocysteine and adenosine. The adenosine is then metabolized to uric acid. The adenosine is also methylated by ADAR to m6A.

There are three primary ways for the epigenetic control of genes: (1) chromatin acetylation deacetylation (i.e., opening or condensation of chromatin); (2) a chronic high fat diet (HFD) causes dysfunctional metabolism and epigenetic folate 1-cabon metabolism (FOCM) of the methyl group of methionine, elevating homocysteine levels (Hcy, characteristic of homocystinuria or HHcy) unequivocally; and (3) miRNA and adenosine deaminase action of RNA (ADAR), m6A, and m5C (Figure 1).

## 2. Background

### 2.1. Diet and Epigenetics

High fat diet (HFD) and metabolic dysfunction patients are three times more likely to die with myocardial infarction and stroke than any other co-morbidity [2,3,4]. Metabolic dysfunction is associated with developing coronary heart disease, hypertension, and heart failure with preserved ejection fraction (HFpEF) [5,6,7]. HFpEF is the hallmark of fatigue, dyspnea, renal insufficiency and volume retention, and exercise intolerance, especially in the elderly and women [8,9,10]. The precise mechanism of HFpEF is unclear. Therefore, identifying the specific effective molecular target for HFpEF therapy is an unmet need. Despite the association of co-morbid conditions, such as hypertension, hypercholesteremia, hyperlipidemia, homocystinuria (HHcy, i.e., homocystinuria, HHcy), diabetes, obesity, and aging, with HFpEF, there is no known direct target for intervention for HFpEF. Homocystinuria is also referred to as an inheritance disorder in homocysteine metabolism causing HHcy, therefore, homocystinuria and HHcy are related.

Interestingly, homocysteine (Hcy) via eNOS pathways contributes to hypertension [11]. The elevated levels of homocysteine (Hcy, i.e., homocystinuria, HHcy) are created by dysregulation in the epigenetic rhythmic methylation/demethylation cycle by folate 1-carbon metabolism (FOCM) and is associated with hypertension [12,13,14,15]. However, the mechanism is unknown.

There is ~1% of methionine (Met) in a HFD containing red meat, which is significantly high for a single amino acid. Red meat, methionine intake, gut dysbiosis, and a threefold mortality risk are also categorical. Quantitative statements require appropriate in primary reference [16].

**Figure 2:**
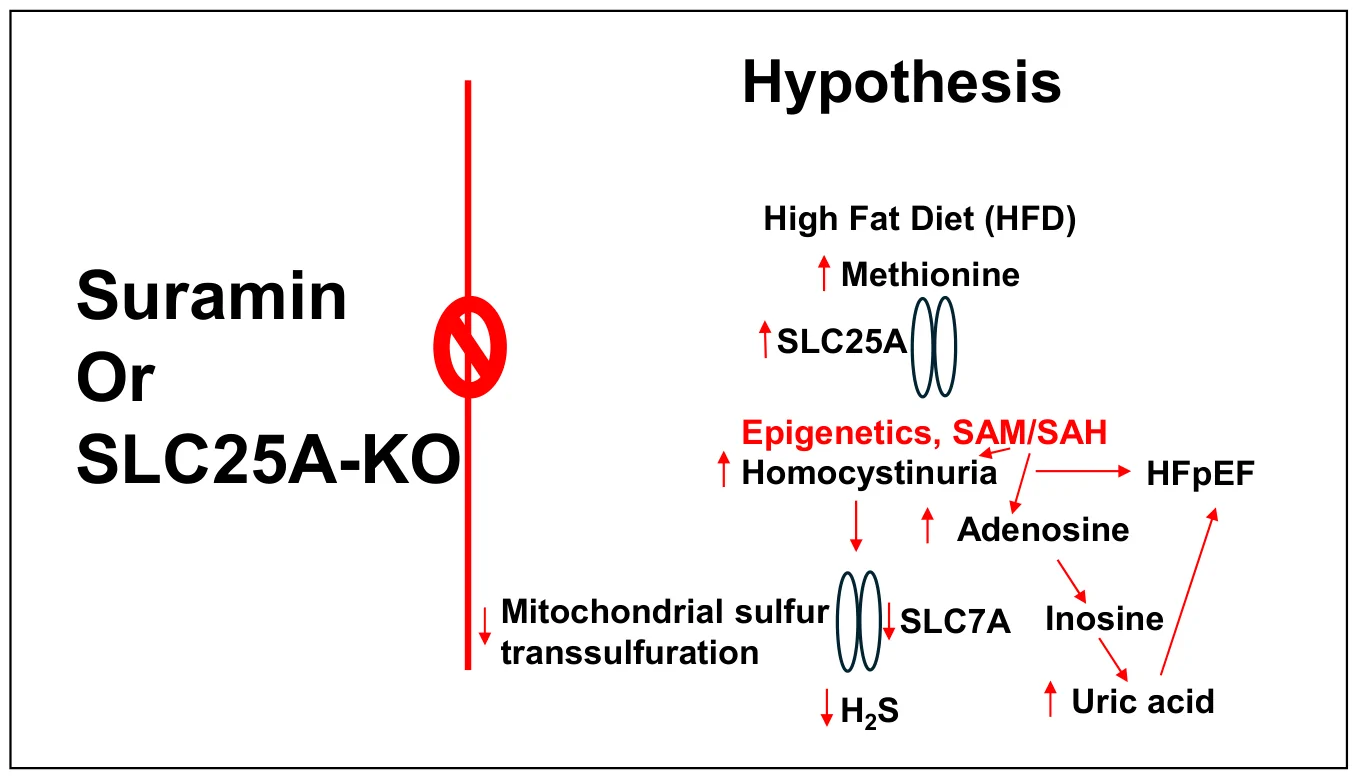
The premise is that dysbiotic HFD increases methionine transporter SLC25A and rhythmic, i.e., methylation of histone lysine by gene writer (DNMT) and eraser (TET), creating hyper homocystinuria (HHcy) and hypertension. The homocysteine transporter SLC7A is decreased, causing disruption in mitochondrial sulfur metabolism and H_2_S levels. The SLC25A knockout, SLC25AKO, or the inhibition of SLC25A by suramin attenuates methionine transport into the cytoplasm, mitigating hypertension and HFpEF.

The study reported that a methionine-restricted diet improves muscle regeneration capacity [17]. Also, methionine restriction mitigates HHcy via FOCM [18]. Therefore, it is novel to block methionine cellular transport (SLC25A) by suramin, mitigate FOCM/HHcy, improve cardiac function, and reverse HFpEF (Figure 2).

Suramin is a pharmacological inhibitor of the mitochondrial transporter SLC25A. However, suramin is a well-known pleiotropic compound that acts as a non-selective antagonist for purinergic receptors (P2X and P2Y) and inhibits various cellular enzymes. We plan to isolate the specific effects on SLC25A from off-target responses in vivo or in vitro. This includes the genetic silence models (e.g., siRNA/shRNA or CRISPR/Cas9 knockout), necessary to validate this target.

A study reported that the inhibition of methyl transferase restores epigenetic metabolism and improves regenerative capacity [17]. Interestingly, methionine is a substrate for epigenetic DNA/RNA/protein, histone methylation, and generates Hcy via the SAM/SAH/SAHH pathways. Hcy is then converted to H_2_S via the transsulfuration CBS/CSE/3MST and transporter SLC7A pathways. HFD, methionine transport, SLC25A activation, FOCM dysregulation, and hyperhomocysteinemia reduced homocysteine transport to the mitochondria via SLC7A/H_2_S transsulfuration signaling and HFpEF.

The HFD via transport and metabolism pathways instigate cardiac remodeling. HHcy is caused by impaired recycling of homocysteine back to methionine by an increase in the epigenetic methylation of H3K4, K9, and H4K20 via the folate 1-carbon metabolism FOCM pathway through an increase in gene writer (DNMT) and decrease in eraser (TET/FTO).

Hyperhomocysteinemia disrupts the DNMT1/TET2 ratio, leading to aberrant histone (H3K4, H3K9, H4K20) and DNA methylation. In the future, the specific gene promoters or loci in cardiomyocytes or cardiac fibroblasts will be mapped to demonstrate how this altered ratio drives cardiac stiffening or loss of compliance.

A HFD causes generational effects [19,20,21] via epigenetic control of the genes. Due to high methionine in a HFD, the gut-dysbiosis instigates de-arrangement in the epigenetic rhythmic methylation/demethylation ratio on DNA/RNA and protein/histones via the DNMT1/TET2 ratio, consequently creating HHcy.

The epigenetic folate 1-carbon metabolism (FOCM) pathway recycles Hcy back to methionine. The transport by SLC25A and epigenetic DNA methylation by FOCM [22,23] is the hallmark of epigenetic memory, i.e., rhythmic gene imprinting and off printing during embryogenesis, development, health, remodeling, and diseases [24]. A single cell RNA sequence analysis revealed an increase in the SLC25A transporter in homocystinuria.

### 2.2. Epigenetics and Cardiac Fibrosis

The premise of this study is also supported by Kay et al. [25,26], and Engler et al. [27,28,29], who revealed the role of transforming fibroblasts and fibrosis in cardiac remodeling. The study by Dees et al. [30] suggests that DNA-methyltransferase 3A (DNMT3A) and DNMT1 in fibroblasts in a SMAD-dependent manner silence the expression of suppressor of cytokine signaling 3 (SOCS3) by promoter hypermethylation. Downregulation of SOCS3 facilitated the activation of signal transducers and activators of transcription 3 (STAT3) to promote fibroblast-to–myofibroblast transition, collagen release, and fibrosis in vitro and in vivo. Re-establishment of the epigenetic control of STAT3 signaling by genetic or pharmacological inactivation of DNMT3A reversed the phenotype of fibroblasts in tissue culture, inhibited TGFb-dependent fibroblast activation, and ameliorated experimental fibrosis in murine models [30,31,32]. Felisbino and McKinsey suggested the role of epigenetics in cardiac fibrosis with an emphasis on inflammation and fibroblast activation [33,34,35]. The authors also demonstrated the role of histone acetylation and reader proteins using small molecule inhibitor of bromo-domain containing protein 4 (BRD4) for mitigating cardiac fibrosis [35]. Interestingly, we demonstrated that Hcy induces collagen expression in a dose- and time-dependent manner [36]. Hcy activates fibroblasts to differentiate into myofibroblasts in murine aortic endothelial cells [37]. Interestingly, most of the human population is mildly HHcy and asymptomatic; however, on a chronic HFD, especially a red meat rich diet that is high in methionine (a substrate for Hcy generation during epigenetically regulated DNA methylation) [23,24], may develop severe HHcy, which will ultimately cause symptomatic HFpEF.

Previously, in humans, we showed the role of a disintegrin and metalloproteinase (ADAM) in connexin-43 (Cx43) degradation in human end stage heart failure, i.e., HFrEF [38]. Others have shown that Cx-43 synchronizes with myocyte-mitochondrial function [39,40]. Here, we focused on the significance of epigenetics via gene writer, eraser, and reader function along with the direct consequences of Hcy in cardiac remodeling and HFpEF.

Inflammation also deserves a dedicated mechanistic paragraph linking metabolic dysfunction, endothelial activation, NF-κB signaling, cytokines, fibroblast activation, and myocardial fibrosis [41].

## 3. Mitochondrial Epigenetics

### Lipid and Homocysteine Theories

According to the homocysteine theory by McCully [42,43], a diet high in animal protein and low in B-vitamins, occur in many foods however are easily destroyed by processing—like meat, cheese, milk, white flour, and canned foods, boxed, refined, processed, or preserved—all are associated with high homocysteine levels. This suggests a strong connection between diet and cardiovascular diseases (CVDs), but one that is different from cholesterol/lipids. Homocysteine theory considers CVDs by protein intoxication. The cholesterol theory (sometimes called the lipid theory) instead demonizes fats. Since proteins and fats often occur in the same foods, the potential dietary treatments for high homocysteine and high cholesterol are similar, with this distinction: an anti-homocysteine diet focuses on what should be eaten, as a preventative, while the anti-cholesterol diet focuses on what should be avoided, as a precipitator. Thus, a diet to lower homocysteine levels would include many natural sources of B-vitamins like fresh fruits and vegetables and would limit animal protein. The cholesterol-reducing diet would limit foods high in saturated fats and cholesterol, like eggs, meat, and butter. The latter is more commercially popular. Here, we propose that an inhibitor of methionine transporter (SLC25A) will reduce the methionine and homocysteine intracellular levels and mitigate HFpEF.

## 4. Conclusions

We conclude that an increase in methionine transporter SLC25A will increase rhythmic methylation/demethylation. This will cause dysfunction in the mitochondrial bioenergetics pathways by epigenetic gene writer (DNMT) and eraser (TET and FTO) ratios [44], creating HHcy and promoting HFpEF. To this end, it is important to use SLC25A-KO, or the inhibitor of SLC25A, suramin, to mitigate HFD-induced epigenetic de-arrangement and HFpEF.

In addition, because homocysteine is transported by the mitochondrial SLC7A transporter and generates H_2_S, it would be very interesting to use transgenic mice over expressing SLC7A and increasing H_2_S production from homocysteine to mitigate HFpEF.

Although lowering the HHcy and treatment with H_2_S reduce obesity and browns the adipose fat [2], the mechanism is unclear. It will be novel to determine for the first time whether a homocysteine mitochondrial transporter (SLC7A) increases mitochondrial sulfur metabolism, transsulfuration, and bioenergetics by H_2_S and mitigates HFD-induced HFpEF.

Also, the role of the kidney is suggested in the development of HFpEF; however, very few studies have demonstrated the contribution of the kidney to HFpEF. Interestingly, the difference between serum and urine metalloproteinases, especially urinary neutrophil gelatinase associated lipocalin (NGAL) in the urine versus serum, will reveal the contribution of NGAL in HFpEF.

Homocystinuria increases blood pressure (via abrogation eNOS pathways) and hypertension. It is unclear whether HFD and homocystinuria contribute to HFpEF. Interestingly, inhibition of DNMT1 mitigated homocysteine-induced hypertension. This suggests that homocysteine, a byproduct of epigenetics, causes hypertension. Also, homocystinuria causes nocturnal hypertension and sudden cardiac deaths. The hypothesis is that a HFD increases methionine transport by SLC25A and induces epigenetic folate 1-carbon metabolism, homocystinuria, and decreases mitochondrial transsulfuration by SLC7A, CBS/CSE/3MST, and H_2_S, causing HFpEF (Figure 2).

Limitations: The limitation is that although several statements, including the proposed HFD–SLC25A–DNMT/TET–SLC7A–H_2_S pathway, are presented as proven causal mechanisms, much of the evidence is indirect, preclinical, or extrapolated from other disease models.

Homocystinuria and hyperhomocysteinemia are not synonymous: homocystinuria generally denotes rare inherited metabolic disorders, whereas hyperhomocysteinemia describes elevated circulating homocysteine from genetic, nutritional, renal, or other causes. This is the distinction between the two terminologies.

SLC25A5a and SLC7A3a, the specific isoforms proposed to transport methionine or homocysteine, respectively, could provide direct supporting evidence and clarify their cellular and mitochondrial localization. This is reported in the Limitations section.

The hypothesis suggests overexpressing the SLC7A transporter in transgenic mice to enhance mitochondrial H_2_S production from homocysteine. In the future, we will perform further studies on physiological safety, transport stoichiometry, or potential systemic toxicity of constitutive mitochondrial amino acid/transporter overexpression for evaluation.

## Data Availability

No new data were created or analyzed in this study. Data sharing is not applicable to this article.

## Abbreviations

CBS: cystathionine beta synthase; CSE, cystathionine gamma lyase; DMG, dimethylglycine; DNMT, DNA methyl transferase; FOCM, folate 1-carbon metabolism; FTO, fat-mass obesity associated protein; Hcy, homocysteine; HHcy, hyperhomocysteinemia; H3K9, histone H3 lysine 9 (methylated gene-off; acetylated gene on); HDAC, histone de-acetylase; HFD, high fat diet; HFpEF, heart failure with preserved ejection fraction; HFrEF, heart failure with reduced ejection fraction; LDH, lactate dehydrogenase; 3-MST, 3-mercaprtopyruvate sulfur transferase; Met, methionine; MMP, matrix metalloproteinase; MTHFS/R, methyl tetrahydrofolate synthase/reductase; OCR, oxygen consumption rate; PC, phosphatidylcholine; PCP, phosphatidylcholine phosphatase; Pi, phosphate; PGC1a, peroxisome proliferator gamma co-activator1alpha; ROS, reactive oxygen species; SAHH, S-adenosine homocysteine hydrolase; SAM, S-adenosine methionine; SAH, S-adenosine homocysteine; SOD, superoxide dismutase; TCA, tricarboxylic acid; TET, ten eleven translocator. TFAM, transcription factor mitochondrial.
